# Effects of different observed datasets on the calibration of crop model parameters with GLUE: A case study using the CROPGRO-Soybean phenological model

**DOI:** 10.1371/journal.pone.0302098

**Published:** 2024-06-13

**Authors:** Yonghui Zhang, Yujie Zhang, Haiyan Jiang, Liang Tang, Xiaojun Liu, Weixing Cao, Yan Zhu

**Affiliations:** 1 School of Computer Engineering, Weifang University, Weifang, P. R. China; 2 Weifang People’s Hospital, Weifang, P. R. China; 3 College of Artificial Intelligence, Nanjing Agricultural University, Nanjing, P. R. China; 4 National Engineering and Technology Center of Information Agriculture, Nanjing Agricultural University, Nanjing, P. R. China; Tennessee State University, UNITED STATES

## Abstract

Suitable combinations of observed datasets for estimating crop model parameters can reduce the computational cost while ensuring accuracy. This study aims to explore the quantitative influence of different combinations of the observed phenological stages on estimation of cultivar-specific parameters (CPSs). We used the CROPGRO-Soybean phenological model (CSPM) as a case study in combination with the Generalized Likelihood Uncertainty Estimation (GLUE) method. Different combinations of four observed phenological stages, including initial flowering, initial pod, initial grain, and initial maturity stages for five soybean cultivars from Exp. 1 and Exp. 3 described in Table 2 are respectively used to calibrate the CSPs. The CSPM, driven by the optimized CSPs, is then evaluated against two independent phenological datasets from Exp. 2 and Exp. 4 described in Table 2. Root means square error (RMSE) (mean absolute error (MAE), coefficient of determination (R^2^), and Nash Sutcliffe model efficiency (NSE)) are 15.50 (14.63, 0.96, 0.42), 4.76 (3.92, 0.97, 0.95), 4.69 (3.72, 0.98, 0.95), 3.91 (3.40, 0.99, 0.96) and 12.54 (11.67, 0.95, 0.60), 5.07 (4.61, 0.98, 0.93), 4.97 (4.28, 0.97, 0.94), 4.58 (4.02, 0.98, 0.95) for using one, two, three, and four observed phenological stages in the CSPs estimation. The evaluation results suggest that RMSE and MAE decrease, and R^2^ and NSE increase with the increase in the number of observed phenological stages used for parameter calibration. However, there is no significant reduction in the RMSEs (MAEs, NSEs) using two, three, and four observed stages. Relatively reliable optimized CSPs for CSMP are obtained by using at least two observed phenological stages balancing calibration effect and computational cost. These findings provide new insight into parameter estimation of crop models.

## 1. Introduction

Crop model is a crucial and powerful tool to reveal the impact of growth conditions change on crop production [1–5]. Crop model uncertainties can be attributed to three main sources, namely model inputs, structures, and parameters [6–9]. Uncertainty in parameters is a crucial factor in model prediction and has attracted significant attention in reducing uncertainty and improving prediction [10–12]. Cultivar-specific parameters (CSPs) are commonly used in crop models to quantify the developmental characteristics that distinguish crop varieties, cultivars, or hybrids. However, since CSPs are unknown and difficult to measure directly in field conditions, estimation of CSPs is of great importance for obtaining reliable model predictions [13]. A natural question is: can more calibration data lead to better results in model parameter estimation and improved model performance? This question is vital for optimizing model parameter estimation, since more optimization data incurs more labor and resources.

Several automatic optimization methods have been developed to calibrate crop model parameters [4,14]. Generalized Likelihood Uncertainty Estimation (GLUE) is a popular Bayesian statistical method for simultaneously calibrating model parameters and estimating model uncertainty [4,15]. He et al. [16] employed GLUE method to estimate the genetic and soil parameters of the CERES-Maize model, achieving accurate prediction of sweet corn production. Jin et al. [17] used GLUE and a formal Bayesian method to optimize the parameters in a simple conceptual model of water balance, and then compared and analyzed the differences between the two methods. Marin et al. [18] utilized GLUE method to optimize ten parameters of the DSSAT-CaneGro model, thereby enhancing its prediction accuracy. With the utilization of GLUE, Pathak et al. [19] estimated the genetic parameters of the cotton model, resulting in improved model performance. Some scholars also employed GLUE to estimate parameters of the crop-hydrology model for enhancing its prediction performance [20]. Sun et al. [21] utilized the GLUE method to evaluate the uncertainty and sensitivity of 25 parameters related to nutrient transport, soil properties, and crop genetics in the agricultural-hydrological model of RZWQM-DSSAT. GLUE has been playing a significant role in calibrating crop model parameters. The incorporation of GLUE into the Decision Support Systems for the Agro-technology Transfer (DSSAT) model has facilitated the estimation of parameters for various crops [13]. The estimation effect of GLUE on model parameters relies on the careful selection and combination of likelihood functions [22]. Recently, GLUE is combined with the parallel processing technique to calibrate the crop model parameters more efficiently and in a shorter timeframe [23]. Most previous studies on GLUE have primarily focused on parameter calibration for various crop models and applied to assess and quantify model uncertainties [24,25], but the quantitative influence of the observed data on model parameter estimation and model performance by using GLUE remains largely unexplored.

In addition, crop phenology controls the life cycle of crops and the partitioning of assimilates between crop organs [26]. Accurate prediction of crop phenology is essential for assessing crop responses to environmental changes and developing management strategies [27–29]. In recent years, CROPGRO-Soybean model has been a commonly used tool for determining the optimal number of experiments [30] and of site × year × planting dates combinations [31] that ensures reliable model calibration and minimizes prediction errors. As a case study of the CROPGRO-Soybean phenological model (CSPM), the objectives of this study are (1) to calibrate the CSPs of CSPM by exploiting different combinations of the four observed phenological stages with the GLUE method; (2) to evaluate and compare the quantitative influence among different combinations of observed phenological stages in estimating CSPs. The results would help to balance the relationships among the amount of observed data, the effect, and the resource in estimating crop parameters.

## 2. Materials and methods

### 2.1 Brief introduction to CROPGRO-Soybean model

The CROPGRO-Soybean model is a process-based dynamic and generic crop model that simulates carbon, water, and nitrogen balances for the soybean plant and soil [32,33]. It includes 15 cultivar-specific parameters that characterize phenology and vegetative and reproductive growth [34,35]. The model operates on a daily time step. It has been embedded in DSSAT and widely used in agriculture production and management [36].

Four critical phenological stages, including initial flowering (FS), initial pod (PS), initial grain (GS), and initial maturity (MS), are simulated in CSPM with the CSPs optimized by GLUE. Three coefficients related to photoperiod sensitivity (CSDL, PPSEN, and R1PPO), and four coefficients related to photothermal duration of life phases (EM-FL, FL-SH, FL-SD, SD-PM) in CSPM are usually calibrated at the cultivar level [37]. These seven coefficients are associated with the simulation of the four phenological stages. The value ranges typical for these parameters are displayed in Table 1 [38].

**Table 1:** Cultivar-specific parameters to be calibrated in CROPGRO-Soybean phenological model.

Symbols	Definition	Range
CSDL	Critical short day (h)	[11.78, 14.6]
PPSEN	Photoperiodic sensitivity (1/h)	[0.129, 0.385]
R1PPO	Increase in daylength sensitivity after anthesis (h)	[0.189, 0.549]
EM-FL	Ideal thermal day from seeding to bloom	[15.5, 28.9]
FL-SH	Time between first flower and first pod	[5.5, 10.0]
FL-SD	Time between first flower and first seed	[12.0, 16.0]
SD-PM	Time between first seed (R5) and physiological maturity	[12.0, 16.0]

### 2.2 Experimental design

Four field experiments (Exp. 1, 2, 3 and 4) were respectively conducted to collect soybean phenological data on five high quality and high yield soybean varieties from the Yangtze-Huai Soybean Breeding Program (Table 2). Row planting was conducted with a length of 2 m, a row spacing of 0.5 m, a planting depth of 3 cm, and with three replicates. In each field experiment, four phenological data of initial flowering (FS), initial pod (PS), initial grain (GS), and initial maturity stage (MS) were recorded according to phenological classification of Fehr et al. [39].

**Table 2:** Detailed information on field experiments.

Experiment	Sowing time	Cultivar-Specific	Location	Latitude and Longitude
Exp. 1	July 5, 2018	Ci (i = 1, 2, 3, 4, 5)	Dangtu of Anhui	31°34’15″N, 118°29’52″E
Exp. 2	June 21, 2019	Ci (i = 1, 2, 3, 4, 5)	Nanjing of Jiangsu	32°3’32″N, 118°37’40″E
Exp. 3	June 21, 2019	Di (i = 1, 2, 3, 4, 5)	Nanjing of Jiangsu	32°3’32″N, 118°37’40″E
Exp. 4	June 24, 2019	Di (i = 1, 2, 3, 4, 5)	Dangtu of Anhui	31°34’15″N, 118°29’52″E

In the field experiments, no obvious light, temperature, water, nutrition, pest, or disease stresses were observed during the crop growth seasons, and the yield of each soybean cultivar is within the normal range. Daily meteorological data were obtained from the meteorological information center of the State Meteorological Administration of China [28]. Actual sowing depth, meteorological data and latitude are used in CSPM. Default values in CSPM for parameters other than CSPs were used.

### 2.3 The research framework and GLUE algorithm

**Fig 1:**
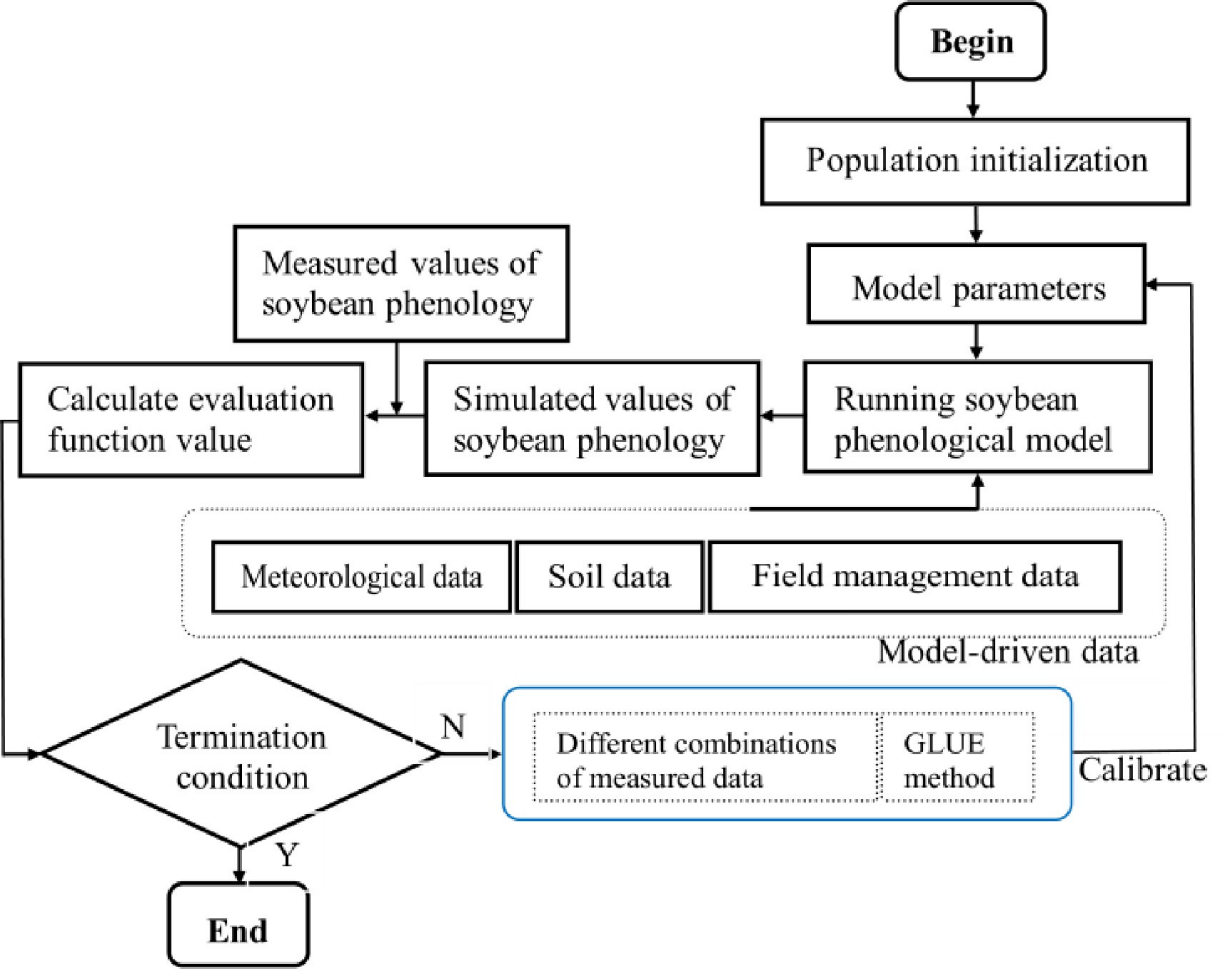
The framework of model parameter estimation based on GLUE and different combinations of observed data in this work.

The framework for estimating model parameters is demonstrated in Fig 1. The steps for using the GLUE method by Beven and Binley [40] is summarized as follows:

**Step 1:** The prior distributions of parameters to be optimized are assumed to be uniform within the ranges of parameter values shown in Table 1. Initial populations composed of parameter combinations are randomly generated with the uniform distribution.
**Step 2:** Each parameter combination is used to drive the CSPM for Monte-Carlo simulation, and the simulation results are employed to compute the related parameters.
**Step 3:** Eq (1) is chosen as the likelihood function in our work.

$$L(\theta_{i}|O_{j}^{t})=1-\frac{Abs(S(\theta_{i})-\bar{O_{j}^{t}})}{\bar{O_{j}^{t}}}$$ (1)

Where *θ*_*i*_ is the *i*^th^ parameter combination composed of CSDL, PPSEN, R1PPO, EM-FL, FL-SH, FL-SD, and SD-PM. *S*(*θ*_*i*_) is the simulated values for the four phenological stages (FS, PS, GS, and MS) calculated using *θ*_*i*_ at time *t. $O_{j}^{t}$* and $\bar{O_{j}^{t}}$are the observed and mean observed value of the *j*^th^ (*j* = 1, 2, 3, 4) phenological stage at time *t*, respectively. $L(\theta_{i}|O_{j}^{t})$ is the likelihood function value corresponding to the *j*^th^ (*j* = 1, 2, 3, 4) phenological stage. *Abs*() is the absolute of a numerical value.

**Step 4:** the combination likelihood function *L*_*cj*_(*θ*_*i*_|*O*_*j*_) corresponding to the the *j*^th^ (*j* = 1, 2, 3, 4) phenological stage is calculated by Eq (2)

$$L_{cj}(\theta_{i}|O_{j})=\overset{N_{ot}}{\underset{\text{t}=1}{\prod }}L(\theta_{i}|O_{j}^{t})$$ (2)

where *O*_*j*_ is the observed value of the *j*^th^ (*j* = 1, 2, 3, 4) phenological stage. *N*_ot_ is the number of observation moments, *N*_ot_ = 1 in this paper.

**Step 5:** The validity of parameter values in crop models is often determined by the prediction accuracy of multiple phenological stages, Eq (3) is employed to calculate the likelihood function of a certain parameter combination.

$$L_{D}(\theta_{i}|O)=\prod_{j=1}^{N_{ps}}L_{cj}(\theta_{i}|O_{j})$$ (3)

Where *L*_*D*_(*θ*_*i*_|*O*) denotes the value of likelihood function corresponding to multiple phenological stages. *N*_ps_ is the number of observed phenological stages. *O* is the observed dataset of phenological stages.

**Step 6**: Eq (3) is used to compute the likelihood function of every parameter combination. The threshold value of the likelihood function is utilized to find out the parameter combinations with “positive effect”. The threshold value is set 0.90, and the parameter combinations corresponding to the likelihood function value greater than 0.90 are considered as having “positive effect” on the simulation results of CSPM. Eq (4) is utilized to obtain the posterior probability density of parameter combination.

$$p(\theta_{i})=\frac{L_{D}(\theta_{i}|O)}{\Sigma_{k=1}^{N_{fi}}L_{D}(\theta_{k}|O)}$$ (4)

where *P*(*θ*_*i*_) is the posterior probability density of *θ*_*i*_. *N*_fi_ is number of parameter combinations filtered with the threshold value of 0.90.

**Step 7:** On the basis of posterior probability density of each parameter combination, Eqs (5) and (6) are used to respectively compute the mean and standard deviation of each variable in the parameter combination.

$$\underset{post}{\hat{\mu }}(\theta_{im})=\sum_{i=1}^{N_{fi}}p(\theta_{i})•\theta_{im},(m=1,\ldots .,7)$$ (5)

where *θ*_*im*_ is the *m*^th^ parameter in *θ*_*i*_. $\underset{post}{\hat{\mu }}(\theta_{im})$ is the mean value of posterior distribution of *θ*_*im*_.

**Step 8:** The array composed of $\underset{post}{\hat{\mu }}(\theta_{im})$(*m* = 1,…,7) is considered as the optimal solution of parameter combination for each calibration. The rest of the notation is the same as above.

**Parameter setting:** During model parameter calibration using GLUE, the number of initial populations is assigned as 20000 and the number of generations is set to 1.

### 2.4 The evaluation criteria

Root means square error (RMSE), mean absolute error (MAE), coefficient of determination (R^2^), and Nash Sutcliffe model efficiency (NSE) are used to evaluate the differences between simulated and measured values.


$$RMSE=\sqrt{\frac{1}{N_{\text{tol}}}\sum_{i=0}^{N_{tol}}{\left(O_{i}-S_{i}\right)}^{2}}$$ (6)

$$MAE=\frac{1}{N_{tol}}\sum_{i=1}^{N_{tol}}Abs(O_{i}-S_{i})$$ (7)

Where, *N*_tol_ is the number of the observed or simulated values. *O*_i_ and *S*_i_ are the observed value and the simulated value, respectively. The lower the value of RMSE (MAE) the better the model performance [41–43].


$$R^{2}=1-\frac{RSS}{TSS}$$ (8)

Where RSS and TSS denote the residual sum of squares and the total sum of squares, respectively. R^2^ has a range between 0 and 1. The values of R^2^ = 1 can be interpreted as “very good” about model performance, values from 0.8 to 1 as “good”, from 0.6 to 0.8 as “satisfactory”, and less than 0.6 as “weak” [41].


$$NSE=1-\frac{\sum_{i=1}^{N_{tol}}{\left(O_{i}-S_{i}\right)}^{2}}{\sum_{i=1}^{N_{tol}}{\left(O_{i}-\bar{O}\right)}^{2}}$$ (9)

Where, *Ō* is the means of observed value. NSE ranges between 0 and 1, the values ranging between 0.75 and 1.0 are considered as “very good” regarding the performance of the model, between 0.65 and 0.75 as “good”, between 0.5 and 0.65 as “satisfactory”, and between 0.4 and 0.5 as “acceptable” [41,43]. The rest of the symbols is the same as above. The definitions of all symbols are given in Table 3.

**Table 3:** List of symbols used in this study.

Symbols	Descriptions
*Abs*()	Absolute value
CSPs	Cultivar-Specific Parameters
CSPM	CROPGRO-Soybean phenological model
MAE	Mean absolute error
NSE	Nash Sutcliffe model efficiency
*N*_fi_	Number of parameter combinations filtered by the threshold value
*N*_ps_	Number of observed phenology phases
*N*_ot_	Number of observing moments of a phenological stage
*N*_tol_	Total number of the observed or simulated values
*O*_i_	Observed value of phenological stage
*Ō*	Mean observed value
RMSE	Root Means Square Error
R^2^	Coefficient of determination
RSS	The residual sum of squares
*S*_i_	Simulated value of phenological stage
TSS	The total sum of squares
*θ*_*i*_	The *i*^th^ parameter combination
*θ*_*im*_	The *m*^th^ parameter of *θ*_*i*_
*S*(*θ*_*i*_)	Simulated value corresponding to parameter *θ*_*i*_
$O_{j}^{t}$	Observed value of the *j*^th^ (j = 1, 2, 3, 4) phenological stage at time *t*
$\bar{O_{j}^{t}}$	Mean observed value of the *j*^th^ phenological stage at time *t*
*O*_*j*_	Observed value of the *j*^th^ phenological stage
$L(\theta_{i}|O_{j}^{t})$	Likelihood function value corresponding to the *j*^th^ phenological stage
*L*_*cj*_(*θ*_*i*_|*O*_*j*_)	Combination likelihood function corresponding to the phenological stage at multiple observing moment
*L*_*D*_(*θ*_*i*_|*O*)	Likelihood function value of multiple phenological stages
*P*(*θ*_*i*_)	Posterior probability density of parameter combination *θ*_*i*_
$\underset{post}{\hat{\mu }}(\theta_{im})$	Mean value of posterior distribution of *θ*_*im*_

### 2.5 Calibration and Evaluation for CSPM

Datasets from Exp. 1 and 3 are used for CSPs estimation and model calibration. Datasets from Exp. 2 and 4 are used for model evaluation (Table 4). CSPs estimation and model calibration for each combination are repeated three times to obtain three sets of optimized CSPs. It is ensured that there are no significant differences among the three sets (P>0.05). The CSPM is then run with these three sets of optimized CSPs to obtain three sets of simulated values of the four phenological stages (FS, PS, GS, and MS). Since there are no significant differences among the three sets (P>0.05), mean value from the three sets of optimized CSPs is considered as the optimum value of the CSPs.

**Table 4:** Data sources for calibration and evaluation.

ID	Method	Calibration data	Evaluation data
1.1	GLUE	Exp.1-Ci-FS	Exp.2-Ci-FS, PS, GS, MS
1.2	GLUE	Exp.1-Ci-PS	Exp.2-Ci-FS, PS, GS, MS
1.3	GLUE	Exp.1-Ci-GS	Exp.2-Ci-FS, PS, GS, MS
1.4	GLUE	Exp.1-Ci-MS	Exp.2-Ci-FS, PS, GS, MS
1.5	GLUE	Exp.3-Di-FS	Exp.4-Di-FS, PS, GS, MS
1.6	GLUE	Exp.3-Di-PS	Exp.4-Di-FS, PS, GS, MS
1.7	GLUE	Exp.3-Di-GS	Exp.4-Di-FS, PS, GS, MS
1.8	GLUE	Exp.3-Di-MS	Exp.4-Di-FS, PS, GS, MS
2.1	GLUE	Exp.1-Ci-FS, PS	Exp.2-Ci-FS, PS, GS, MS
2.2	GLUE	Exp.1-Ci-FS, GS	Exp.2-Ci-FS, PS, GS, MS
2.3	GLUE	Exp.1-Ci-FS, MS	Exp.2-Ci-FS, PS, GS, MS
2.4	GLUE	Exp.1-Ci-PS, GS	Exp.2-Ci-FS, PS, GS, MS
2.5	GLUE	Exp.1-Ci-PS, MS	Exp.2-Ci-FS, PS, GS, MS
2.6	GLUE	Exp.1-Ci-GS, MS	Exp.2-Ci-FS, PS, GS, MS
2.7	GLUE	Exp.3-Di-FS, PS	Exp.4-Di-FS, PS, GS, MS
2.8	GLUE	Exp.3-Di-FS, GS	Exp.4-Di-FS, PS, GS, MS
2.9	GLUE	Exp.3-Di-FS, MS	Exp.4-Di-FS, PS, GS, MS
2.10	GLUE	Exp.3-Di-PS, GS	Exp.4-Di-FS, PS, GS, MS
2.11	GLUE	Exp.3-Di-PS, MS	Exp.4-Di-FS, PS, GS, MS
2.12	GLUE	Exp.3-Di-GS, MS	Exp.4-Di-FS, PS, GS, MS
3.1	GLUE	Exp.1-Ci-FS, PS, GS	Exp.2-Ci-FS, PS, GS, MS
3.2	GLUE	Exp.1-Ci-FS, PS, MS	Exp.2-Ci-FS, PS, GS, MS
3.3	GLUE	Exp.1-Ci-FS, GS, MS	Exp.2-Ci-FS, PS, GS, MS
3.4	GLUE	Exp.1-Ci-PS, GS, MS	Exp.2-Ci-FS, PS, GS, MS
3.5	GLUE	Exp.3-Di-FS, PS, GS	Exp.4-Di-FS, PS, GS, MS
3.6	GLUE	Exp.3-Di-FS, PS, MS	Exp.4-Di-FS, PS, GS, MS
3.7	GLUE	Exp.3-Di-FS, GS, MS	Exp.4-Di-FS, PS, GS, MS
3.8	GLUE	Exp.3-Di-PS, GS, MS	Exp.4-Di-FS, PS, GS, MS
4.1	GLUE	Exp.1-Ci-FS, PS, GS, MS	Exp.3-Ci-FS, PS, GS, MS
4.2	GLUE	Exp.3-Di-FS, PS, GS, MS	Exp.4-Di-FS, PS, GS, MS

ID.

Here i = 1, 2, 3, 4, 5. Exp.1-Ci-FS, PS, GS, MS denotes the four observed phenological data of initial flowering (FS), initial pod (PS), initial grain (GS), and initial maturity stage (MS) from Ci in Exp. 1. Exp.3-Di-FS, PS, GS denotes the three observed phenological data including FS, PS, and GS from Di in Exp. 3. Meanings for the other symbols follow this pattern.

## 3. Results

**Fig 2:**
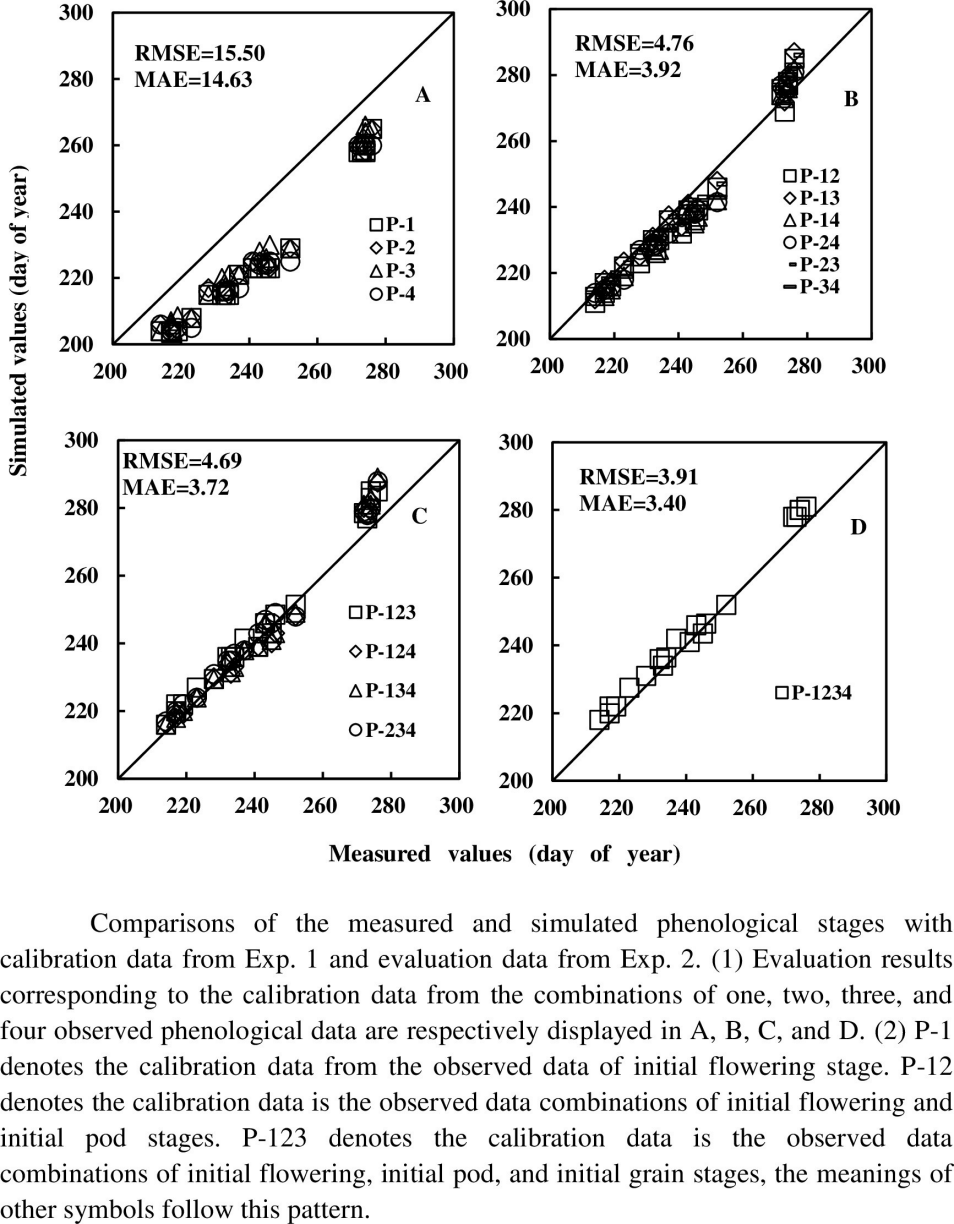
Comparisons of the measured and simulated phenological stages with calibration data from Exp. 1 and evaluation data from Exp. 2.

**Fig 3:**
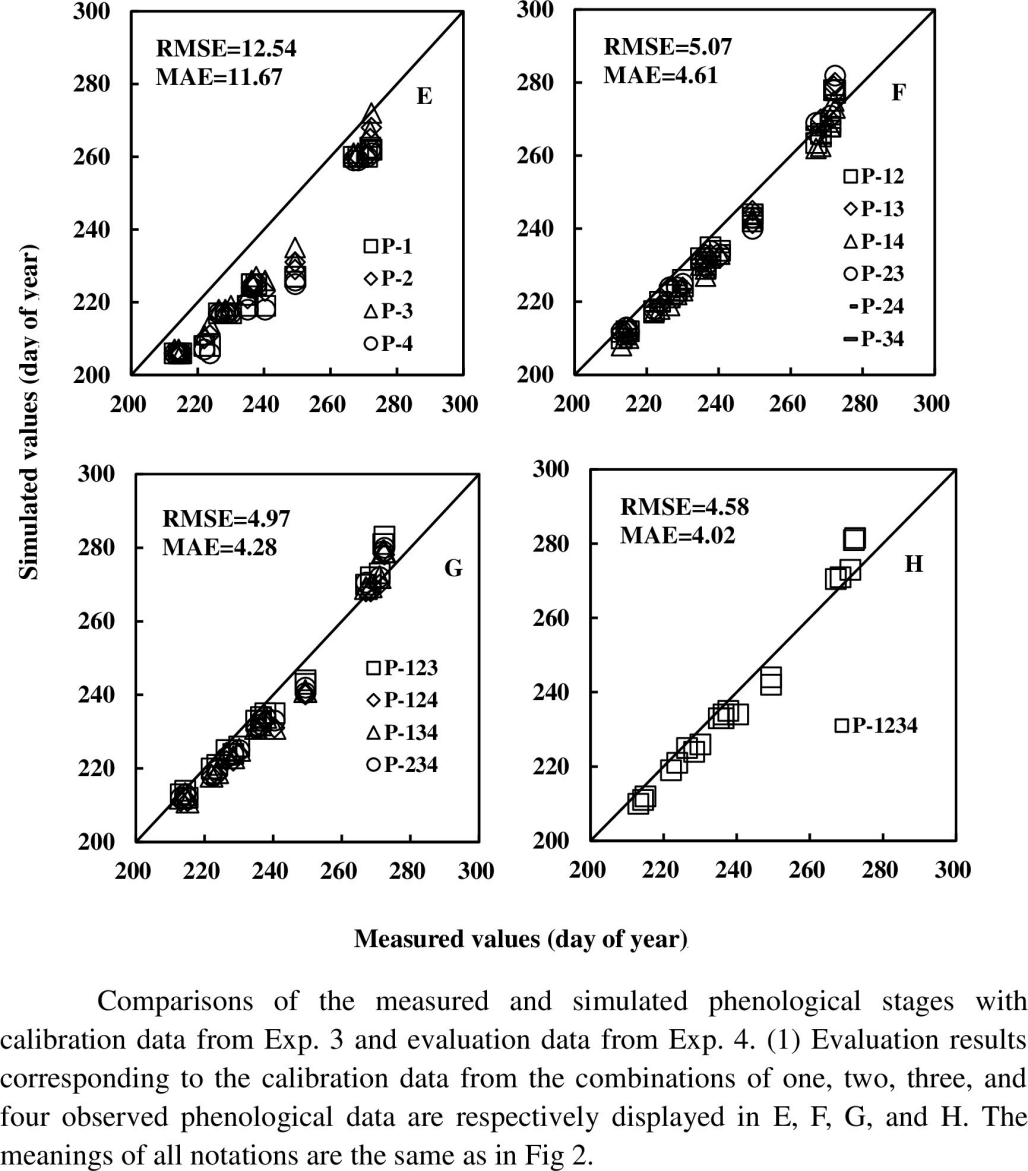
Comparisons of the measured and simulated phenological stages with calibration data from Exp. 3 and evaluation data from Exp. 4.

**Fig 4:**
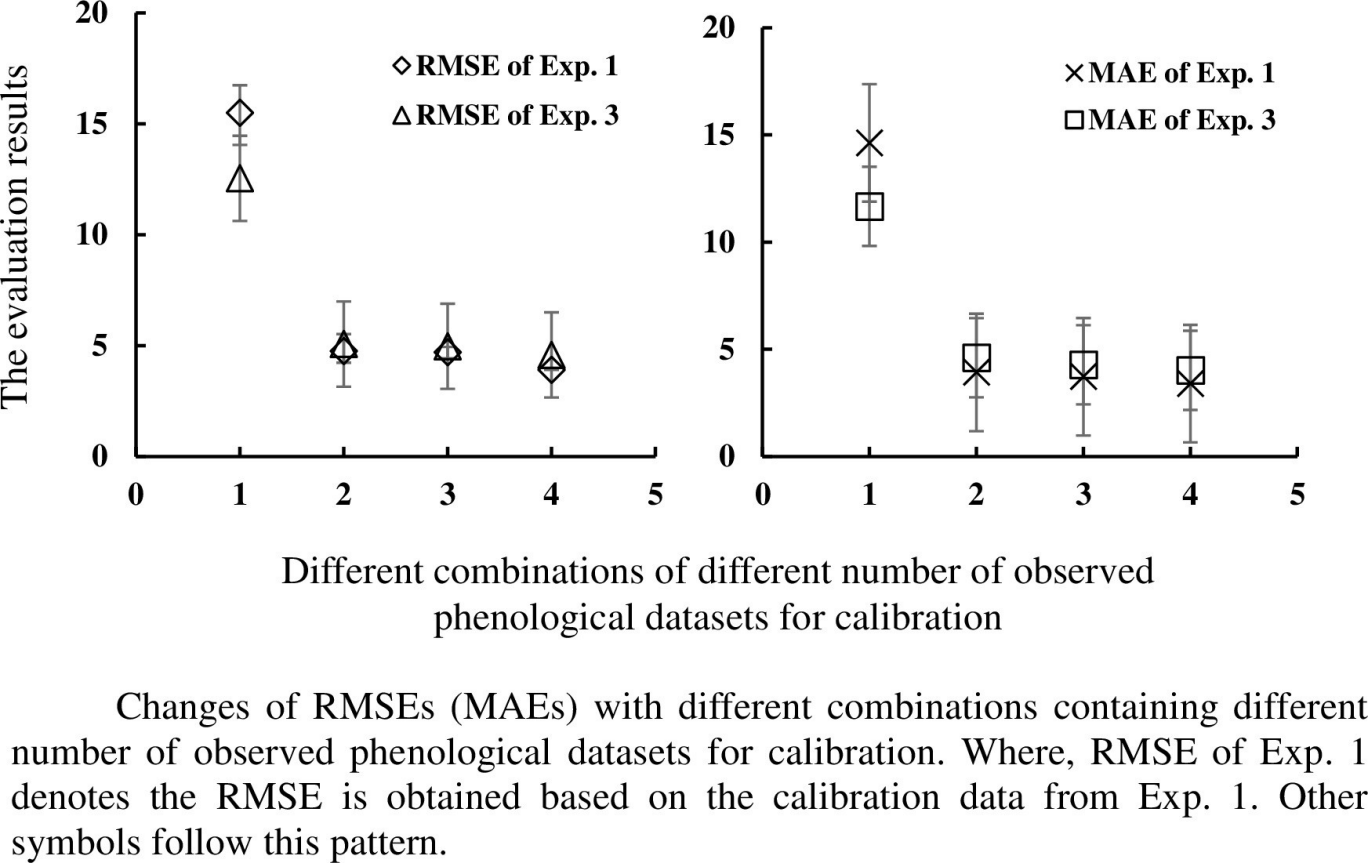
Changes of RMSEs (MAEs) with different combinations containing different number of observed phenological datasets for calibration.

**Fig 5:**
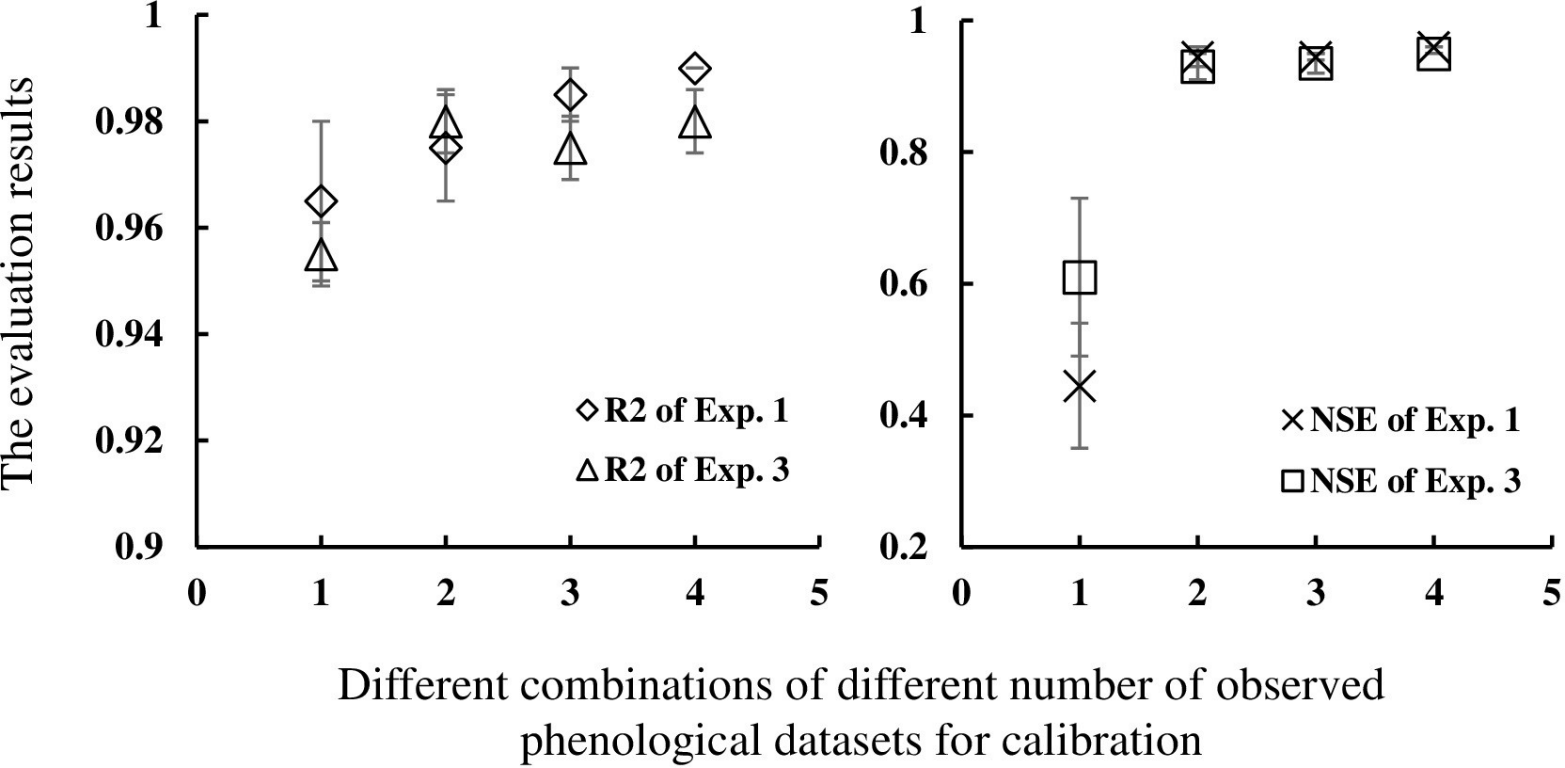
Changes of R^2^ (NSEs) with different combinations containing different number of observed phenological datasets for calibration. Where, R^2^ of Exp. 1 denotes the R^2^ is obtained based on the calibration data from Exp. 1. Other symbols follow this pattern.

The RMSE, MAE, R^2^, and NSE values for each combination and cultivar are shown in Table 5. Average RMSE(MAE, R^2^, NSE) values are 15.50 (14.63, 0.96, 0.42), 4.76 (3.92, 0.97, 0.95), 4.69 (3.72, 0.98, 0.95), and 3.91 (3.40, 0.99, 0.96) for the calibration datasets from Exp. 1 and validation datasets from Exp. 2; and 12.54 (11.67, 0.95, 0.60), 5.07 (4.61, 0.98, 0.93), 4.97 (4.28, 0.97, 0.94), and 4.58 (4.02, 0.98, 0.95) for the calibration datasets from Exp. 3 and validation datasets from Exp. 4. The simulated and measured values are displayed in Figs 2 and 3. Based on results in Figs 4 and 5, it can be observed that the RMSE (MAE) value decreases, and R^2^ (NSE) increases with an increase in the number of observed phenological stages used for parameter calibration.

**Table 5:** Evaluation results of the calibration data from the different observed data combinations in Exp.1 and Exp. 3.

Results	P-1	P-2	P-3	P-4	P-12	P-13	P-14	P-23	P-24	P-34	P-123	P-124	P-134	P-234	P-1234
RMSE of Exp. 1	16.74	15.88	14.05	16.59	4.99	4.52	5.52	4.23	4.70	4.50	4.90	4.43	4.94	4.47	3.91
MAE of Exp. 1	16.35	15.40	13.58	16.08	4.25	3.63	4.75	2.93	4.10	3.88	4.08	3.38	3.73	3.70	3.40
R^2^ of Exp. 1	0.97	0.97	0.97	0.96	0.97	0.97	0.97	0.98	0.98	0.98	0.98	0.98	0.98	0.99	0.99
NSE of Exp. 1	0.35	0.41	0.54	0.36	0.94	0.95	0.93	0.96	0.95	0.95	0.94	0.95	0.94	0.95	0.96
RMSE of Exp. 3	13.39	11.96	10.33	14.16	4.52	5.13	5.85	4.86	5.18	4.79	4.49	5.47	5.04	4.83	4.58
MAE of Exp. 3	12.56	11.28	9.61	13.23	4.30	4.52	5.53	4.12	4.78	4.45	3.67	4.83	4.39	4.24	4.02
R^2^ of Exp. 1	0.95	0.96	0.97	0.94	0.98	0.98	0.99	0.97	0.99	0.99	0.98	0.97	0.97	0.98	0.98
NSE of Exp. 1	0.54	0.63	0.73	0.49	0.95	0.93	0.91	0.94	0.93	0.94	0.95	0.92	0.94	0.94	0.95

P-1 denotes the calibration data is the observed data of initial flowering stage. P-12 denotes the calibration data is the observed data combinations of initial flowering and initial pod stages. P-123 denotes the calibration data is the observed data combinations of initial flowering, initial pod, and initial grain, and the meanings of other symbols follow this pattern. RMSE of Exp. 1 denote the average RMSE corresponding to the calibration data from Exp. 1, and the meanings of other symbols follow this pattern.

There are four, six, four and one class combinations of calibration data containing one, two, three, four observed phenological datasets. For the four-class data combination scenario, RMSE (MAE, R^2^, NSE) falls within the range of [14.05, 16.74] ([13.58, 16.35], [0.96, 0.97], [0.35, 0.54]), [4.23, 5.52] ([2.93, 4.75], [0.97, 0.98], [0.93, 0.96]), [4.43, 4.94] ([3.38, 4.08], [0.98, 0.99], [0.94, 0.95]), and [3.91, 3.91] ([3.40, 3.40], [0.99, 0.99], [0.96, 0.96]) for the calibration datasets and evaluation datasets respectively from Exp. 1 and Exp. 2, and [10.33, 14.16] ([9.61, 13.23], [0.94, 0.97], [0.49, 0.73]), [4.52, 5.85]([4.12, 5.53], [0.97, 0.99], [0.91, 0.95]), [4.49, 5.47]([3.67, 4.83], [0.97, 0.99], [0.92, 0.95]), [4.58, 4.58] ([4.02, 4.02], [0.98, 0.98], [0.95, 0.95]) for those from Exp. 3 and Exp. 4. No other obvious difference of calibration effects is found for the different combinations with the same number of observed phenological stages.

## 4. Discussions

Several recent studies emphasize the importance of quantifying model uncertainty components in crop models [8,44,45]. The contribution of parameters to model uncertainty has been studied and compared in relation to the structures of model inputs [9,46,47]. Parameter uncertainty arises from the uncertainty of model parameter values [48]. This uncertainty can be attributed to the data used for calibration, the limited amount of data available, and the calibration process itself [4]. Therefore, quantifying the impact of different calibration data on estimating crop parameters is vital to minimize uncertainty and improve model performance. But research in this area is largely unexplored. This work aims to fill the knowledge gap. we collect the phenological data of five soybean cultivars from four field experiments to calibrate model parameters using the GLUE method and evaluate the CSPM performance. We utilize fifteen different combinations of the four observed phenological stages for each cultivar to calibrate the CSPs of CSPM. By comparing and analyzing the calibration effects between different combinations, we can identify the rules for balancing calibration effect and computational cost in estimating crop parameters. Identifying the minimum number of observed datasets needed for model calibration will allow rational use of available resources and increase the efficiency on agriculture research.

The evaluation results show that using a larger number of observed stages for model calibration result in the lowest RMSE (MAE) and largest R^2^ (NSE). Combinations of observed data using one, two, three, and four phenological stages to calibrate model can provide R^2^ ranging from 0.95 to 0.99, with “good” effect regarding the performance of the model; those using two, three, and four phenological stages provide NSE ranging from 0.91 to 0.96, with “very good” effect; but those using one phenological stage just provide NSE less than 0.73 (Table 5). R^2^ is insensitive as an evaluation parameter in this work. The above analysis shows that the combinations using two, three, and four phenological stages provide similar results in RMSE (MAE, NSE) with no significant differences among them in this work. Similar results were found in other studies: generally, with the number of experiments used to calibrate the model increases, the RRMSE becomes smaller, and the best cost-benefit during the calibration was two experiments [28]. This phenomenon suggests that one set of phenological stage alone cannot adequately represent the genetic characteristics of a crop cultivar. Rather, a comprehensive collection of phenological stages can provide a better reflection of the cultivar’s genetic traits. The results indicate relatively reliable CSPs is obtained by using two or three observed phenological stages, taking into consideration both model performance and computation cost. These results would provide a variety of options of observed data in the parameter calibration of crop phenological model according to the actual conditions with the good calibration effect, which can reduce the limitations of only using the data of flowering and physiological maturity stages in previous researches [27,28]. The quantitative relationship between calibration performance and the number of datasets holds for other crops remains to be further investigated.

The major contributions and novelty of this study are summarized as follows: (1) The quantitative influences of different combinations of four observed phenological stages in soybean on calibrating the CSPs of CSPM are explored using GLUE method. (2) Comparing and analyzing the calibration effects between different combinations using different evaluation indicators of RMSE, MAE, R^2^, and NSE, can help us to identify the rules for balancing calibration effect and computational cost in estimating crop parameters. (3) The results would provide decision to obtain the suitable observed data according to the actual conditions and reduce the limitations of previous researches.

## 5. Conclusions

The GLUE method is used in this study to explore the quantitative influences of different combinations of observed phenological stages on calibrating the CSPs. Fifteen combinations of observed phenological stages from each soybean cultivar are used to estimate the CSPs of CSPM. Furthermore, independently measured data are employed to evaluate the CSPM model (Table 4). The evaluation results indicate that as the number of observed phenological data in calibration increases, the calibration effect also increases (Table 5 and Figs 4 and 5), with minor differences among the calibration combinations of two, three, and four observed phenological data. Relatively reliable CSPs for the CSPM considering the cost-benefit relation are obtained using at least two observed phenological stages.

These results can serve as guidance for studying crop model calibration, which balances model accuracy and ground-truth data collection. For future research, more observed phenological data of soybean plants need to be collected under various conditions, such as different planting dates, climate regions, nutrient levels, and water availability, in order to further verify our conclusions and to discover new rules for estimating crop model parameters.

## Supporting information

S1 File (XLSX)
